# Analysing protein complexes in plant science: insights and limitation with AlphaFold 3

**DOI:** 10.1186/s40529-025-00462-2

**Published:** 2025-05-22

**Authors:** Pei-Yu Lin, Shiang-Chin Huang, Kuan-Lin Chen, Yu-Chun Huang, Chia-Yu Liao, Guan-Jun Lin, HueyTyng Lee, Pao-Yang Chen

**Affiliations:** 1 Institute of Plant and Microbial Biology, Academia Sinica, Taipei, 115 Taiwan; 2 Institute of Plant Biology, National Taiwan University, Taipei, 106 Taiwan; 3 Bioinformatics Program, Institute of Statistical Science, Taiwan International Graduate Program, Academia Sinica, Taipei, 115 Taiwan; 4 Bioinformatics Program, Taiwan International Graduate Program, National Taiwan University, Taipei, 115 Taiwan; 5 Genome and Systems Biology Degree Program, Academia Sinica and National Taiwan University, Taipei, 115 Taiwan

**Keywords:** AlphaFold 3, Crop resilience, Protein structure, Protein–protein interaction, Protein complex, Structure biology

## Abstract

AlphaFold 3 (AF3), an artificial intelligence (AI)-based software for protein complex structure prediction, represents a significant advancement in structural biology. Its flexibility and enhanced scalability have unlocked new applications in various fields, specifically in plant science, including improving crop resilience and predicting the structures of plant-specific proteins involved in stress responses, signalling pathways, and immune responses. Comparisons with existing tools, such as ClusPro and AlphaPulldown, highlight AF3’s unique strengths in sequence-based interaction predictions and its greater adaptability to various biomolecular structures. However, limitations persist, including challenges in modelling large complexes, protein dynamics, and structures from underrepresented plant proteins with limited evolutionary data. Additionally, AF3 encounters difficulties in predicting mutation effects on protein interactions and DNA binding, which can be improved with molecular dynamics and experimental validation. This review presents an overview of AF3’s advancements, using examples in plant and fungal research, and comparisons with existing tools. It also discusses current limitations and offers perspectives on integrating molecular dynamics and experimental validation to enhance its capabilities.

**Supplementary Information:**

The online version contains supplementary material available at 10.1186/s40529-025-00462-2.

## Introduction

**Fig. 1:**
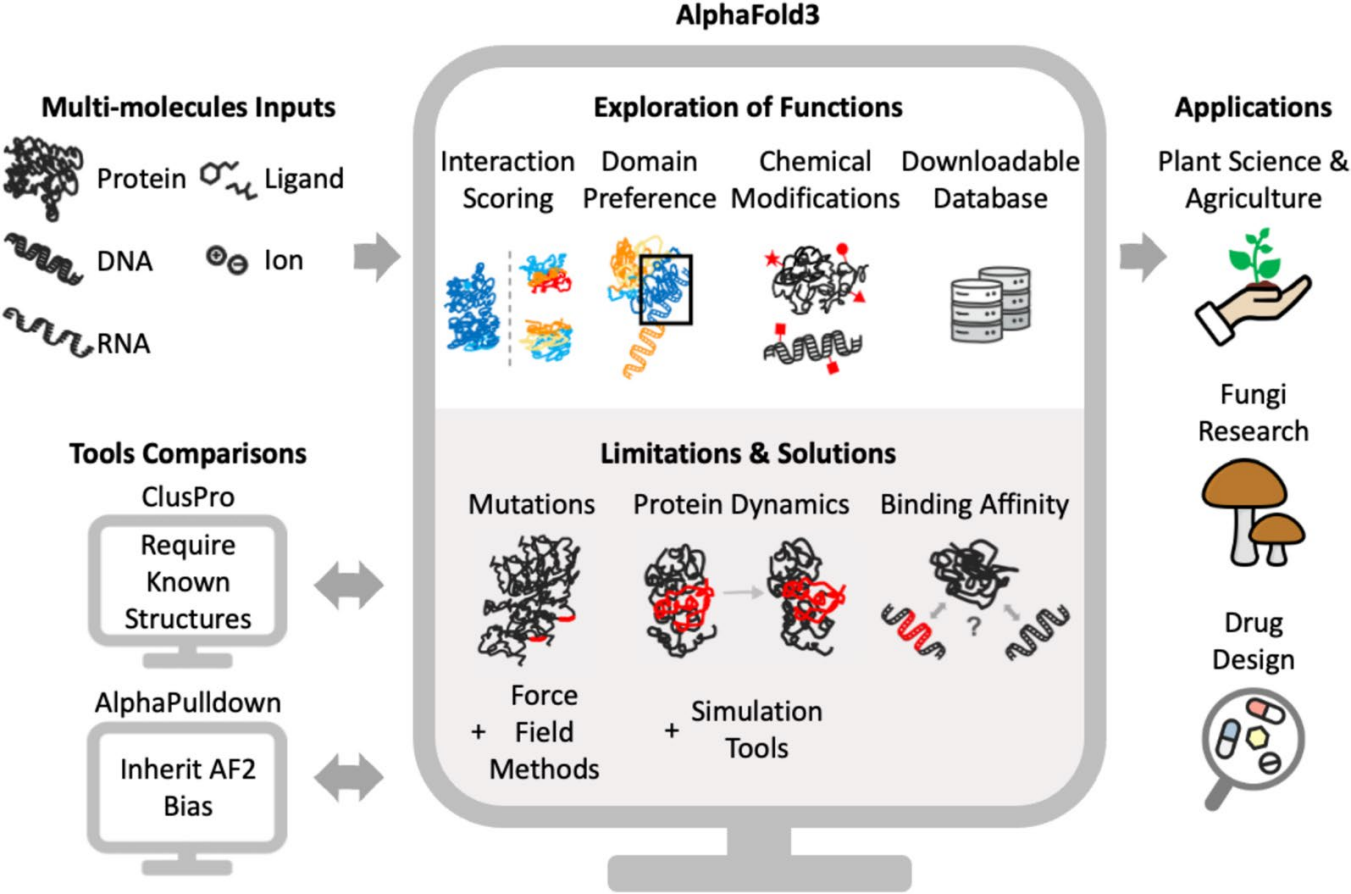
Overview of analysing protein complexes using AlphaFold 3 (AF3) in plant sciences. The left panel includes the multi-biomolecule inputs and comparisons of ClusPro and AlphaPulldown. The middle upper panel is the functions expiring, including the ability to distinguish between known and poor protein interactions, domain preference, structural changes of chemical modification, and the downloadable database, while the lower is limitations and potential solutions in predicting mutation-induced structural change, dynamic protein behaviours, and predicting protein-DNA binding affinity. The right panel is the potential application of AF3

Proteins are essential molecules that drive all cellular processes in plants and other organisms, from growth and development to stress responses and immune signalling (Bao 2009). Their three-dimensional (3D) structures provide insights into functions, missense variations, and interactions (Akdel et al. 2022). Predicting such protein complex structures could provide significant insight for unravelling cellular mechanisms. However, accurately modelling these biological complexes has long been a significant challenge due to the complexity of protein–protein interactions, limited structural data for many plant proteins, and the inefficiency of the traditional computation tools in predicting large multi-molecular assemblies (Bryant et al. 2022). This challenge has been addressed by AlphaFold 3 (AF3 Webserver, https://alphafoldserver.com/welcome), an advanced machine-learning tool, offers precise predictions of 3D protein structures and their interactions, enabling a wide range of applications in crop improvement and drug design (Fig. 1) (Abramson et al. 2024). AF3 expands its capabilities in speed, high accuracy, and the modelling of joint structures in complexes involving multi-molecules. Its accuracy reached nearly 75% for all tested protein–protein interactions, about 10% higher than existing tools, based on known protein structures in the Protein Data Bank (PDB). With these improvements, AF3 is poised to significantly accelerate progress in structural biology, drug discovery, and synthetic biology (Desai et al. 2024).

This review presents an overview of AF3’s advancements with examples from plant and fungal research, comparing it to existing tools. It also examines current limitations and discusses potential improvements through molecular dynamics and experimental validation.

### From AlphaFold 2 to AlphaFold 3

AlphaFold 2 (AF2) revolutionised structural biology in predicting protein 3D structures directly from amino acid sequences (Akdel et al. 2022). It significantly outperformed other prediction tools, such as DynaMut2 (Rodrigues et al. 2021) and FoldX (Schymkowitz et al. 2005), when compared to experimental techniques such as X-ray crystallography and cryo-electron microscopic (cryo-EM). AF2 achieved higher structural accuracy in CASP14 (Critical Assessment of Structure Prediction) (Jumper et al. 2021). Unlike FoldX, which relies on energy-based calculations (Schymkowitz et al. 2005), AF2 uses deep learning to capture complex structural features for better prediction.

Building on the success of AF2, AF3 provides several notable advancements and new technologies capable of high-accuracy prediction of complexes in nearly all molecular types present in the PDB, which contains known 3D molecule structures derived from experimental results (Berman et al. 2000). Table 1 outlines four key differences between AF2 and AF3. One improvement in AF3 is the replacement of a specific AF2 module (Evoformer) with a simpler module (Pairformer) in AF3, which significantly reduces the computational burden of multiple sequence alignment (MSA) processing (Abramson et al. 2024). AF3 employed a smaller and simpler MSA embedding block by reducing the number of blocks to four and using pair-weighted averaging method for MSA representation. For instance, in AF2, predicting a multi-protein complex (~ 3000 residues) required aligning thousands of sequences, consuming extensive GPU memory and computational time. In contrast, AF3’s Pairformer eliminates this requirement, allowing the same structure to be predicted in a fraction of the time and reducing memory usage by approximately 30%. Additionally, AF3 replaces AF2's structure module with a new Diffusion module that directly predicts raw atomic coordinates instead of using amino-acid-specific frames and side-chain torsion angles. These changes improve data efficiency and extend AF3's applicability to a wider range of chemical structures.

**Table 1:** Comparisons of AF2 and AF3

	AF2 (Akdel et al. 2022)	AF3 (Abramson et al. 2024)
Purpose	Predicts single protein structures	Predicts interactions and joint structures for multi-molecular complexes
Key Module	Evoformer module for MSA-based predictions	Pairformer module without heavy reliance on MSA
Input Types	Single protein sequences	Proteins, nucleic acids, small molecules, ions, and modified residues
Advancements	Focused on evolutionary information through MSAs	Diffusion-based modelling and raw atomic coordinate predictions

MSA: multiple sequence alignment

We consider AF2 to be a gold standard for single-protein structure predictions using MSA, whereas AF3 is specifically designed for modelling interactions between proteins and other molecular entities (Abramson et al. 2024). In our opinion, reduced reliance on MSA makes AF3 better suited for predicting protein–protein interactions, protein–nucleic acid complexes, and assemblies involving small molecules or ions.

### The inputs and outputs of AlphaFold 3

AF3 accepts inputs such as post-translational modifications, protein interaction partners, partial structural data derived from experiments, and annotations about the cellular environment, including lipid bilayer context for membrane proteins or roles in large molecular complexes, enabling more precise predictions.

**Fig. 2:**
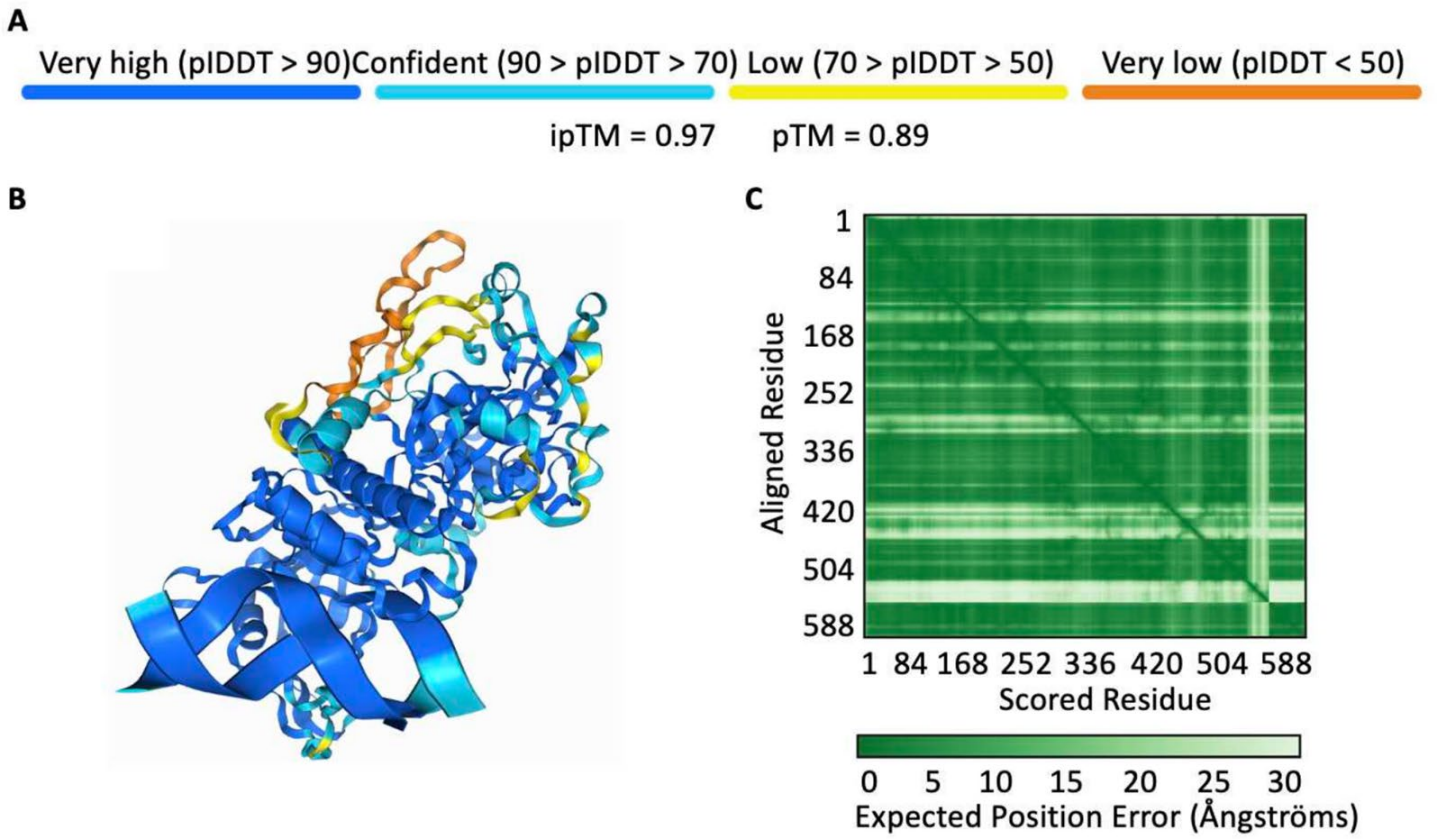
AlphaFold Server output interface. **A** The ipTM score and pTM score of the model, representing the confidence of prediction. **B** A 3D structure with the highest rank among five predicted models, the regions are coloured according to pLDDT scores. **C** A PAE matrix that shows the confidence in relative positions of residue pairs. The example protein for demonstration is Arabidopsis thaliana histone methyltransferase SUVH6 in complex with methylated DNA (PDB ID: 6A5N)

AF3 is available on the AlphaFold Server (AlphaFold Server 2024) or via GitHub repository (google-deepmind 2024), which generates five predictions per seed, showing the top-ranked one on the result page (Supplementary Note 1). Key outputs of AF3 as demonstrated in Fig. 2, include.

1. the predicted 3D structure which ranked the highest among the five predicted models;
2. two predicted scores, predicted template modelling (pTM) and interface predicted template modelling (ipTM); and
3. predicted aligned error (PAE) matrix heatmap.

The 3D structure is coloured based on the predicted local distance difference test (pLDDT) for residue-level confidence. pTM and ipTM are both confidence scores, the former is used to assess the reliability of the overall protein (complex) structure while the latter is used to evaluate the interactions between protein subunits, respectively. The PAE matrix helps represent possible positional errors between residues, with lower PAE values corresponding to higher pTM or ipTM scores.

### Applications of AlphaFold 3

AF3 has been used to address several long-standing challenges across multiple scientific fields by providing deeper insights into complex biological mechanisms.

In agriculture, improving crop resilience is a critical priority. Plant proteins are essential for key physiological processes such as photosynthesis, disease resistance, and stress tolerance. Understanding their 3D structures precisely would provide critical insights into enzyme functions, signalling pathways, and protein–protein interactions, which are crucial for improving crop growth, enhancing yield, and increasing resilience to environmental stressors. Structural knowledge allows for the targeted modification of proteins involved in drought tolerance, pest resistance, and nutrient efficiency, leading to the development of more climate-resilient and high-yielding crop varieties. However, most experimentally resolved plant protein structures come from model species like Arabidopsis thaliana, while less than 1% of protein sequences in major crops have been structurally characterised (Ceasar and Ebeed 2024). This gap limits functional research and the ability to apply structural insights to crop improvement, slowing progress in agricultural biotechnology.

AF3 offers a promising solution to bridge this gap, especially to enhance our understanding of crop resilience under environmental stress. For example, small heat shock proteins (sHSPs) are crucial for preventing protein misfolding and aggregation during heat stress in plants (Waters et al. 1996; Wu et al. 2022). These proteins have been shown to enhance heat and salt tolerance in different organisms by preventing thermal aggregation of crucial enzymes. Moreover, transgenic rice plants overexpressing HSP20 exhibited enhanced root growth and higher germination rates under heat and salinity stress, therefore maintaining protein function and cellular integrity under stress conditions (Guo et al. 2020). Such large protein complexes traditionally required labour-intensive experiments to resolve. AF3 can computationally predict the structure of these complexes, which will shed light on their binding sites and downstream target proteins, ultimately aiding the development of heat-tolerant crops. For example, carbohydrate-binding sites have been predicted on large protein datasets by combining AlphaFold Multimer and binding interface deep-learning tools (He et al. 2024), demonstrating the practicality of structural modelling of protein complexes.

Structure-based drug design, which optimises drug chemical structures using target protein structures (Sliwoski et al. 2014), is expected to be greatly influenced by AF3. AF2 has been integrated into drug discovery engines with successful examples (Merz et al. 2010; Ren et al. 2023), and AF3 offers higher-resolution structural predictions essential for identifying catalytic sites, drug-binding pockets, and active site flexibility. This advancement is particularly impactful for protein families lacking existing structural data, accelerating the discovery and optimisation of therapeutic compounds.

### Insights and limitations

This review focuses on AF3 advancements, applications, comparisons with existing tools, and limitations alongside potential solutions (see Fig. 1 for an overview), providing researchers with insights for effective use. We highlight AF3’s impact on plant sciences, particularly in agriculture, bioengineering, and structural biology, by accurately predicting complex protein–protein and protein–nucleic acid interactions. We compared AF3 with existing tools such as ClusPro (Kozakov et al. 2017) and AlphaPulldown (Yu et al. 2023) to highlight their respective strengths and unique capabilities. Lastly, we discussed the limitations of AF3, particularly in predicting large molecular assemblies, dynamic protein behaviours, and interactions involving proteins from underrepresented species in the PDB, and suggested potential solutions. Our review offers guidance on AF3 and identifies areas for future improvement in plant sciences and innovation in structural biology.

## Exploration of AlphaFold 3 functions

AF3 opens up wider applications for structure prediction of multi-molecular complexes. In this section, we use experimentally validated cases of protein interactions, in plants and fungi, to showcase AF3’s capability in identifying protein–protein interactions. In particular, AF3 demonstrated higher prediction accuracy when using specific domain sequences rather than full-length sequences. In addition, AF3 can also accurately predict chemically modified nucleic acids and protein structures, serving as an important analytical tool for studying structural and functional changes caused by chemical modifications. In our opinion, the AlphaFold Protein Structure Database (AFDB, Abramson et al. 2024) offers a vast collection of 3D-annotated protein structures, which may provide for those who need large-scale structural analysis and advancing protein-related research.

### Distinguishing between known and poor protein interactions

Accurate prediction of protein–protein interaction is the main feature of AF3. One of the prediction scores ipTM is expected to indicate confidence of interaction, where above 0.8 is high confidence and below 0.6 is failed prediction (Abramson et al. 2024). We explore whether ipTM indeed differs between an experimentally validated interaction versus non-interacting elements and whether this difference is visually obvious through the 3D structures of predicted complexes.

**Fig. 3:**
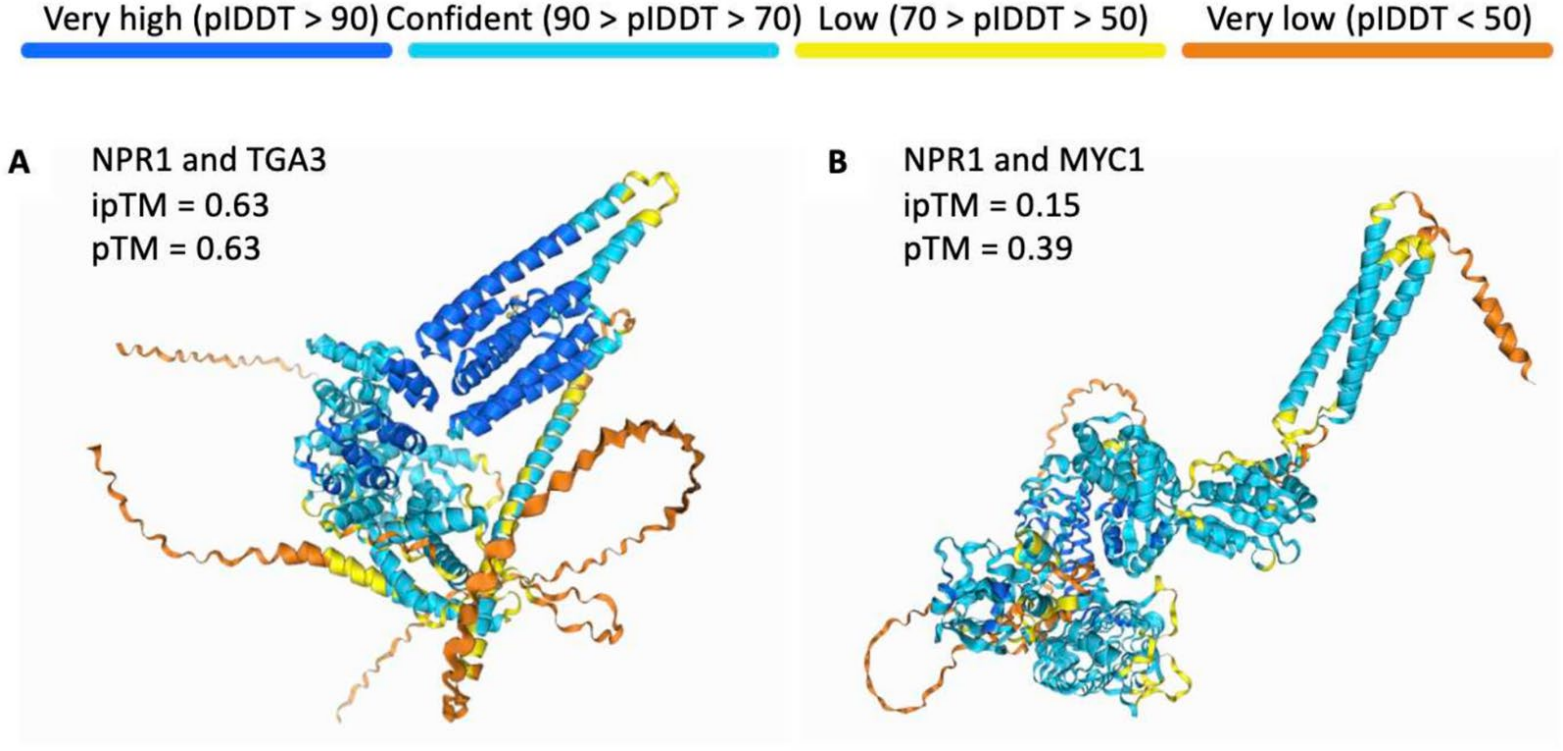
NPR1-TGA3 and NPR1-MYC1 complexes predicted by AF3. **A** Positive test case for protein complex with known interaction, NPR1 and TGA3. **B** Negative test case for proteins that do not interact, NPR1 and MYC1

We examined AF3’s accuracy in predicting interactions between key plant proteins, the master regulator of plant immune signal NPR1 and transcription factor TGA3 in *Arabidopsis thaliana* (Kumar et al. 2022). Figure 3A shows the predicted complex with both ipTM and pTM scored 0.63. Inspection of known interacting sites in the predicted complex shows that hydrophobic residues of NPR1 are correctly projected onto the concave structure of TGA3, as depicted in Figure S1A. Out of all seven residues of NPR1 that are known to form hydrogen bonds or salt bridges with residues of TGA3, only one was identified in AF3 prediction, as shown in Figure S1B. The lack of accurate bonds predicted might explain why the ipTM and pTM scores are only of average confidence. As a comparison, another transcription factor with no known interaction, MYC1, was used to replace TGA3. Unlike TGA3 that has a basic leucine zipper motif (Miao et al. 1994) and regulates plant immunity, MYC1 belongs to the basic helix-loop-helix family and is involved in root hair development that has no known role in plant immunity (Zhao et al. 2012). Their structural and functional differences make MYC1 unlikely to interact with NRP1. AF3 predicted the NPR1-MYC1 complex to have scores below 0.4 (Fig. 3B), with the ipTM score being particularly low (0.15), suggesting poor interaction. The 3D structures of these two test cases differ visually in terms of folding and colours which indicate the local structure confidence estimate (pLDDT), though the differences observed are inconclusive in determining the interaction quality. In short, ipTM score can clearly indicate the quality of protein–protein interactions. A similar conclusion is drawn for protein–ligand interactions. Protein sequences that do not bind ATP involve recognising the absence of specific ATP-binding motifs, such as the Walker A and Walker B motifs (Chauhan et al. 2009). There is only a 0.2 ipTM score of the predicted result.

With these test cases from plant research, we are able to confirm that even though the AF3 model will attempt to find interactions between two input molecules, the prediction scores can clearly indicate their interaction likelihood. This demonstrates that AF3 functions as a robust complement to the experimental determination of protein interactions that are usually labour-intensive.

### Domains are preferred over full-length sequences

Protein interaction occurs through specific sites, where the amino acids on one surface of a protein structure form attractive bonds with the surface of another protein, ligand or DNA (Weiner et al. 1982). These surfaces are termed binding domains, where portions of sequences form distinct structural units for binding and are usually evolutionarily conserved (Marsden and Orengo 2008). As protein functions are commonly studied via domains as subunits (Lee and Lee 2009), it is important to ask whether the predicted scores differ when inputting the sequence of whole protein versus domain regions only.

Using a known receptor-kinase pair that mediates brassinosteroid signalling in plants (Li et al. 2002; Nam and Li 2002) as a test case, we extracted full-length protein sequences and sequences of interacting domains for Brassinosteroid insensitive protein (BRI1) and kinase BAK1. In Table 2, we showed that both ipTM and pTM scores of full sequence input are only nearly half of the scores when interacting domains are used. Since the interaction of these proteins was proven experimentally by affinity chromatography (Li et al. 2002) and coimmunoprecipitation (Nam and Li 2002), the ipTM score is expected to be higher than 0.8 to indicate high confidence. The low scores obtained using full-length inputs therefore failed to reflect the true binding affinity.

**Table 2:** AF3 prediction scores of interactions between BRI1 and BAK1 by inputting full-length sequences and interacting domain only

	ipTM	pTM	Length of BRI1 (aa*)	Length of BAK1 (aa)
Full-length sequences	0.38	0.45	1196	615
Interacting domains	0.90	0.91	24	97

The interacting domain position for BRI1 is 727 to 750, and BAK1 is 67 to 163

*aa: amino acid

It shows that inputting interacting domains returns higher accuracy in prediction than full-length sequences. This phenomenon has been reported in single-structure prediction, where small protein fragments obtain about 5% higher sensitivity and specificity than full-length proteins (Lee et al. 2024). It is hypothesised that the disordered regions in full-length proteins may contribute to variations in prediction accuracy in AF2. Our test suggests that this characteristic persists in AF3, influencing protein–protein interaction prediction and emphasising the importance of selecting appropriate input sequences for optimal results.

To conclude, domain sequences are recommended over full-length proteins as AF3 input. Information on protein domains can be easily obtained from protein family databases such as the InterPro consortium (Blum et al. 2024) or predicted de novo through algorithms such as HMMER (Eddy 2009) for enhancing the accuracy of protein structure and interaction predictions. By focusing on well-defined domains, researchers can bypass challenges associated with the variability and flexibility of full-length proteins.

### Detecting structural changes of chemical modification

AF3 allows the detection of structural changes caused by chemical modifications. Chemical modifications of nucleic acids and proteins affect their structure and biological function (Wang and Vasquez 2023). Nucleic acids can be modified in various ways, including methylation, carboxylation, oxidation, and formylation; while proteins can be modified through phosphorylation, acetylation, methylation, citrullination, etc. Different modifications display different physicochemical properties (Mann and Jensen 2003). For instance, citrulline modification of arginine in a protein causes loss of positive charge and therefore partial unfolding of the protein (Tarcsa et al. 1996). Additionally, acetylation of a protein’s lysine residue can neutralise its positive charge, weaken histone-DNA interactions, and promote a more open chromatin structure, thereby enhancing gene expression (Pandey and Gayen 2024).

Such modifications have been linked to specific biological functions (Luscombe et al. 2000; Dantas Machado et al. 2022). For example, a circadian clock protein of the fungus *Neurospora* is progressively phosphorylated for structural change, which then commits the protein for degradation (Querfurth et al. 2011). Yet, the actual conformational changes caused by these modifications are not well understood. A systemic study of glycosylation and phosphorylation in 276 proteins showed only 7% and 13% undergo global structural changes, respectively (Xin and Radivojac 2012). This indicates that the effect of chemical modification on protein structure may be subtle, further emphasizing the need for close examination.

In AF3, protein backbone prediction accuracy was increased by 20% when various types of chemical modifications were modelled (Abramson et al. 2024). This greatly supports the efforts in understanding chemical modifications through pinpointing small or spatially-localised structural changes of nucleic acids and proteins, which will further shed light on changes in biological functions.

### AlphaFold protein structure database as a downloadable resource

AFDB (AlphaFold Protein Structure Database, https://alphafold.ebi.ac.uk) is a collection of more than 200 million entries of 3D protein structure prediction based on amino acid sequences (Varadi et al. 2024), including those from model organisms *Arabidopsis thaliana*. It provides a searchable and downloadable resource for 3D annotation of protein structures without queuing for new predictions through the AlphaFold Server.

Each entry contains basic information about the protein, such as integration from other data resources like UniProt accession ID (The UniProt Consortium 2007) and known biological function. The visualisation of the predicted 3D protein structure with pLDDT score and PAE matrix for assessing inter-domain accuracy is also included. The last section lists similar structures found in PDB. Using 3D superposition, homologous proteins can be identified with higher accuracy particularly when sequence-based homology inference is weak (Illergård et al. 2009). For example, querying the *Arabidopsis thaliana* downy mildew resistance protein AT1G58602 results in another disease resistance protein in tomato NRC1. This allows annotation of evolutionary relationships between proteins as well as species.

The two limitations of AFDB (as of version September 2024) are that (1) protein–protein interaction predictions are not included since AFDB is based on AF2; and (2) if the query protein is novel with no previous information in the database, no results will be returned. To conclude, AFDB offers a catalogue of pre-calculated 3D structures that is particularly useful for large-scale analysis and protein evolution studies.

## Comparative performance with other tools

To better understand AF3’s advancements, we compared its performance with two pioneering tools ClusPro (Kozakov et al. 2017) and AlphaPulldown (Yu et al. 2023) (Table 3). These tools represent established methods for protein–protein interaction prediction, each with distinct algorithms and specialised features. Our comparisons highlight their strengths, limitations, and unique applications in structural biology research.

**Table 3:** Comparison of AlphaFold 3, ClusPro, and AlphaPulldown for protein interaction prediction

	AlphaFold 3	ClusPro	AlphaPulldown
Algorithm	Diffusion-based deep learning	Rigid-body docking	AlphaFold-Multimer integration
Scoring and Ranking	Reports confidence scoring which prioritises interaction candidates	Ranks clustered models by energy conformation and reports top-ranked model	Reports confidence scoring which prioritises interaction candidates
Input Requirements	Protein sequences, nucleotide sequences, small molecules, and ions	Pre-determined 3D structures	Protein sequences
Applicable on	Any biomolecules combination and not limited to pairs	Protein–protein pair	Protein–protein pair

### ClusPro predicts based on known structures

ClusPro is a popular computational tool to predict protein–protein interaction. It requires predetermined protein structures as input and employs rigid-body docking algorithms for predicting interactions, which rely on protein shape complementarity and surface compatibility. It then filters out models with unfavourable electrostatic interactions or poor solubility, groups similar results, and presents the top-ranked candidates from each group (Brenke et al. 2012; Kozakov et al. 2013, 2017; Vajda et al. 2017; Desta et al. 2020; Jones et al. 2022; Zhao et al. 2024). Unlike ClusPro, which requires only known 3D protein structures as input, AF3 begins with amino acid sequences and predicts individual protein structures before modelling their interaction (Desai et al. 2024; Abramson et al. 2024; Wee and Wei 2024).

As some proteins undergo substantial conformational changes upon binding, ClusPro is unable to capture these adaptations since its prediction relies on fixed 3D structures. Another limitation of ClusPro is that it only predicts protein–protein pairs, while AF3 can handle multiple proteins and interact with other biomolecules (Wester et al. 2003; Ramakers et al. 2017; Shemesh et al. 2021; Sweeney et al. 2023). For example, AF3 accurately modelled the structure of fatty acid-binding proteins (FABPs) and identified the binding pockets where the fatty acid ligands interact with these receptors, highlighting its potential in analysing protein–ligand interactions (Nam 2024). Its adaptability to handle protein flexibility and diverse biomolecular interactions makes it a versatile tool for addressing complex structural biology questions.

### AlphaPulldown inherit the bias from AF2

AlphaPulldown is a specialised tool designed for protein–protein interaction studies (Yu et al. 2023). It integrates AlphaFold-Multimer’s, an extension of AF2, predictive power with experimental data to streamline protein–protein interaction screening and modelling (Evans et al. 2021). AlphaPulldown provides functionalities such as confidence scoring and visualisation, enabling the identification and prioritisation of protein interaction candidates. This makes it particularly valuable for researchers working on interactomes or validating experimental protein–protein interaction data, especially when studying higher-order oligomeric complexes (Mosalaganti et al. 2022). By combining computational predictions with experimental evidence, AlphaPulldown bridges the gap between structural biology and interactomics before AF3 is released.

However, AlphaPulldown inherits limitations from its AF2-derived architecture, notably biases from MSA-based modules (Abramson et al. 2024). This bias arises from the reliance on evolutionary information, which can lead to inaccuracies when homologous sequences are limited. In contrast, AF3 reduces reliance on MSA to efficiently predict protein–protein interactions and offers greater flexibility for diverse biomolecular structures. Overall, AF3 provides broader versatility and user-friendly accessibility via its web server, while AlphaPulldown excels in precision and experimental integration for protein–protein interactions but requires more computational resources and expertise.

## Limitation of AF3 and potential solutions

AF3 achieves high accuracy by improving its deep-learning architecture from AF2 and expanding its training data (Abramson et al. 2024). It still faces challenges in several aspects, particularly in handling large protein complexes with mutations, modelling protein dynamics, and dealing with binding affinities between proteins and substrates (Desai et al. 2024; Abramson et al. 2024; Wee and Wei 2024). Here we analyse these limitations and suggest potential solutions.

### Predicting mutation-induced structural change

Even small mutations in specific residues can greatly impact the overall folding conformation of proteins, affecting their interactions and functions (Jubb et al. 2017). Traditionally, scientists need to undergo time-consuming purification steps before working on examining the impact of mutations on structures and interactions.

**Fig. 4:**
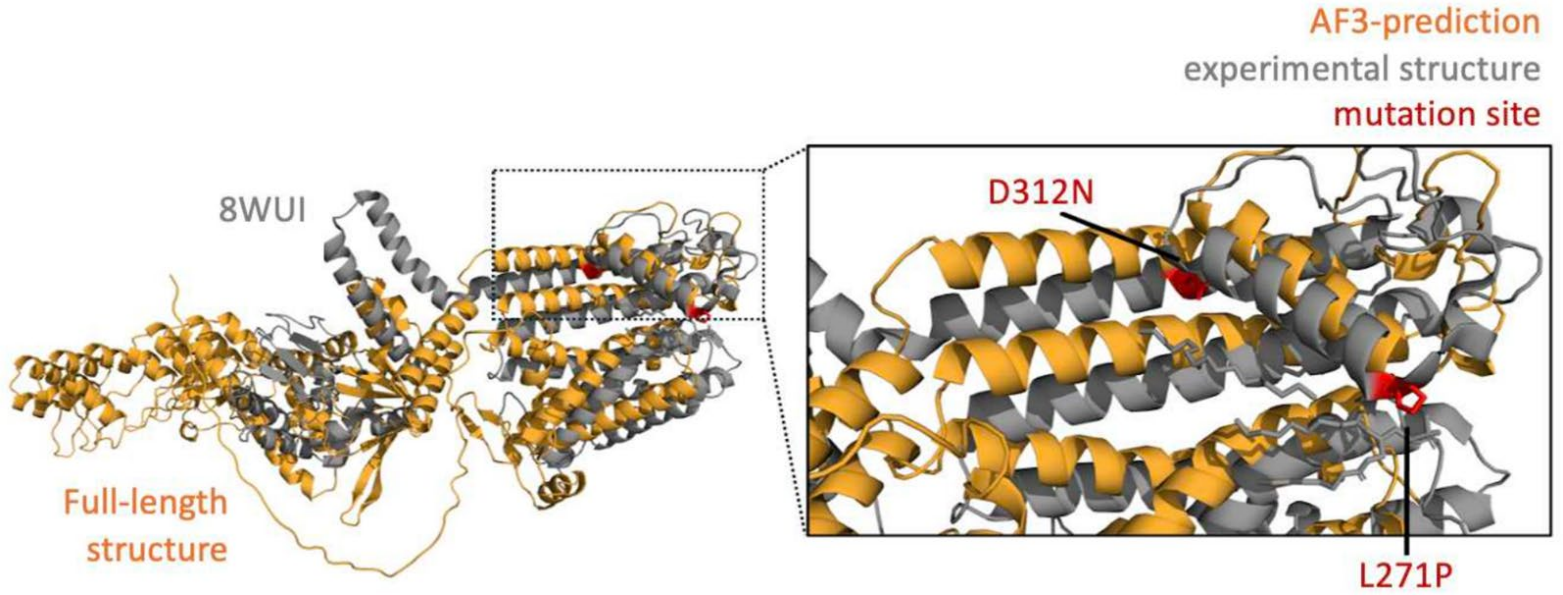
Structure alignment between AF3 predicted models and PDB model of point mutated protein. SKOR full-length structure is in orange while the experimental structure of SKOR mutant is in grey structure (PDB ID: 8WUI). The double mutation sites (D312N/L271P) are highlighted in red. The structures are aligned and visualised using PyMOL

Although AF3 has shown its potential ability to detect mutation-induced structural changes, its accuracy remains inconsistent. A recent study highlighted a strong correlation (Pearson correlation coefficient = 0.86) between AF3 and results from experimental validation (Wee and Wei 2024) in binding free energy (BFE) change, a metric for alteration of binding affinity caused by mutation (Moal and Fernández-Recio 2012). These results are not universally applicable in our test set. For instance, when AF3-predicted structure of *Arabidopsis thaliana* potassium channel SKOR D312N/L271P double mutant was aligned to cryo-EM structure (PDB ID: 8WUI, published on April 10, 2024) using PyMOL v3.0 (Yuan et al. 2017; Schrödinger 2024). It displayed low similarity (Fig. 4, Supplementary Note 2), suggesting that AF3 may struggle to accurately predict mutation-induced structural changes in certain cases.

Even a seemingly insignificant single-point mutation can cause significant changes in protein folding as well as their functions (Wu et al. 1999). Improving the prediction of these mutation-induced changes would be helpful. It has been shown that AF3 is weak in predicting mutational effects on protein structure, especially when coevolutionary signals are weak or absent (Lu et al. 2024). To overcome AF3’s inability to capture atomic-level interactions, traditional all-atom force field methods may be able to complement this AF3 limitation by simulating the physical interactions between atoms in a protein complex. The methods can detect precise atomic-level conformational changes, enabling them to capture mutation-induced effects on structure and binding affinities. Combining AF3’s efficient multi-mutation protein structure predictions with force field-based simulations of atomic-level changes improves scalability and precision, offering deeper insights into how mutations affect protein structure and function.

### Consideration of protein dynamics

Protein dynamic refers to the range of movements and conformational changes that proteins undergo, driven by intrinsic properties and environmental factors. It includes stochastic fluctuations of chemical bonds between atoms and rotation of residues and domains, which play a critical role in protein function. Environmental factors, such as high temperature or high acidity (low pH), can significantly impact these dynamics (Fraser et al. 2011; Deller et al. 2016; Ellaway et al. 2024). For example, increased temperature causes structural movements in the catalytic regions of protein cytosolic malate dehydrogenase (cMDH), altering its enzymatic activity (Dong et al. 2018). Similarly, the visual opsin, a group of proteins made light-sensitive via a chromophore, adopts its active conformation only at low pH (Goncalves et al. 2010), demonstrating the functional importance of environmental influences. However, AF3 predicts only static protein structures under neutral, stable conditions, limiting its ability to evaluate environmental impacts on protein dynamics (Pak et al. 2023; Niazi et al. 2024).

Given AF3’s current limitations in modelling protein flexibility, integrating molecular dynamics (MD) simulations can refine predicted structures and enhance accuracy. MD simulations incorporate environmental factors such as ligand interactions and solvent effects, providing conformational flexibility that AF3 alone cannot achieve. Protein complexes can be simulated under specific conditions by GROMACS (Van Der Spoel et al. 2005) and AMBER (Salomon‐Ferrer et al. 2013). For example, AF3-predicted structures can serve as starting points for MD simulations to model ligand-induced conformational shifts in enzyme active sites or to explore structural changes in response to pH variations in membrane proteins. With the help of these MD simulators, we can obtain a deeper understanding of protein flexibility and dynamic changes in different environmental conditions.

### Predicting protein-DNA binding

DNA-binding proteins play crucial roles in gene regulation, where their binding preferences on DNA sequences determine their biological functions. AF3 has been shown to predict protein-nucleic complexes with higher accuracy than other tools such as RoseTTAFold2NA (Baek et al. 2024). However, it still faces challenges due to the complexity of sequence-specific interactions, electrostatic forces, and chromatin context, which current computational models do not fully account for Abramson et al. (2024).

**Fig. 5:**
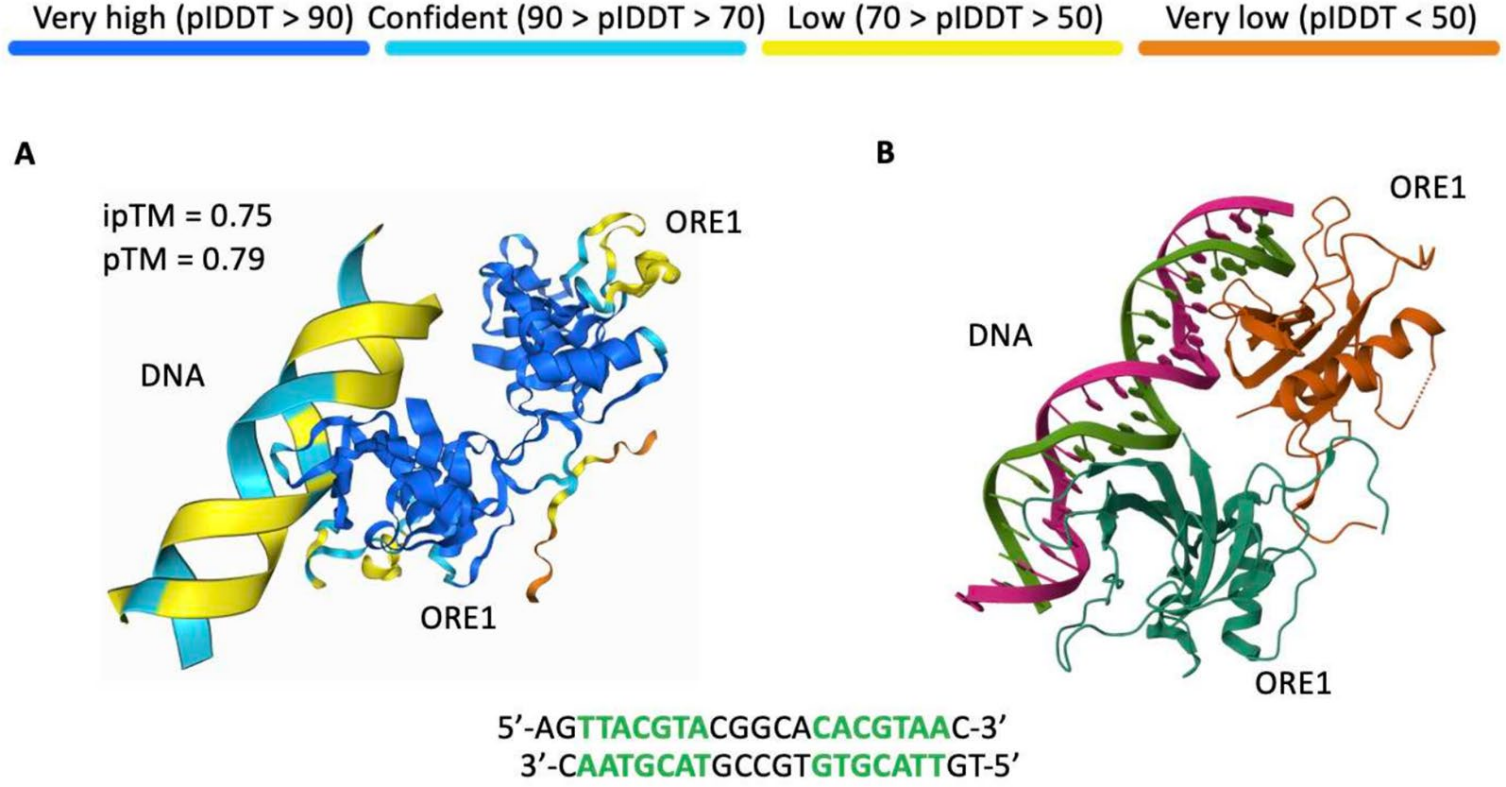
ORE1-DNA interaction complexes predicted by AF3. **A** AF3-predicted complex and **B** PDB model (PDB ID: 7XP3) of ORE1 and DNA segments containing ORE1-specific binding motifs (green sequences). DNA segments and protein structure are represented by ribbon plots. The sequences of double stranded DNA segments are represented at the bottom of the plots. The colours of the ribbons in **A** represent pLDDT scores

We used AF3 to predict interactions between the ORE1 transcription factor’s DNA-binding domain and DNA in *Arabidopsis thaliana*. This structure of this complex was previously validated with X-ray diffraction (Chun et al. 2023) and deposited in PDB (PDB ID: 7XP3) as our benchmark. As shown in Fig. 5A, AF3 successfully identified the DNA-binding domain on ORE1, which is similar to the PDB benchmark (Fig. 5B). However, the DNA motif that is bound by ORE1 is incorrectly predicted by AF3.

AF3 lacks motif-specific recognition capabilities. To address this, motif-based search algorithms such as MEME (Bailey et al. 2009) or experimental techniques like chromatin immunoprecipitation (ChIP-seq) can provide critical complementary data. These methods can identify statistically significant motifs, uncovering potential binding sites predicted by AF3 domains, while ChIP-seq provides empirical evidence of binding sites. Combining these approaches can more accurately determine DNA-binding preferences, addressing AF3's current limitations in this field.

To sum up, AF3 offers advantages in terms of speed and scalability, but may not always capture the full complexity of protein interactions in physiological conditions (Ward et al. 2022). Traditional experimental methods such as pull-down assays and yeast two-hybrid (Y2H) are still fundamental and necessary to support the predicted results.

## Conclusions

AF3 greatly improves the speed and accuracy of complex protein structure prediction by incorporating simpler modules (*i.e.*, Pairformer and Diffusion). With the growing adoption, more and more biologists are convinced that it will continue to change the way biological research is conducted, particularly in addressing key challenges in agriculture. Therefore, we believe that an in-depth exploration of the functions and applications of AF3 will be beneficial for researchers, enabling them to use AF3 more effectively.

AF3 has demonstrated excellent accuracy in predicting multi-molecular complex structures, especially when using domain sequences. This is particularly relevant in plant biology, where understanding protein complexes involved in stress responses, such as immune signalling pathways. Furthermore, AF3 provides the ability to analyse chemical modifications of biomolecules, becoming an important tool for investigating how these modifications influence protein structure and function. Additionally, AFDB further supplies a large amount of 3D protein structure data, which can provide researchers with large-scale structural analysis. These features make AF3 a popular choice for wide use in multiple biological research fields, including plant sciences and drug design. Moreover, compared with the existing ClusPro and AlphaPulldown, which predict the protein–protein interaction tools, AF3 provides a more user-friendly interface and broader applicability, making it a preferred choice for researchers studying plant-specific processes such as nutrient transport or signalling.

Although AF3 has brought many innovations, it still faces challenges in certain complex biological problems, such as the structure prediction of mutants in full-length proteins, which are common in plant enzymes and regulatory proteins. These challenges can be addressed by integrating force field-based and deep learning approaches to provide more reliable predictions. For the prediction of protein dynamics, it is recommended that the AF3 be combined with software that specifically models how protein complexes adapt to their environments, thereby improving the accuracy of protein structure predictions in specific environments. Furthermore, although AF3 cannot directly predict DNA binding affinity, its ability to predict protein-DNA interaction sites has been greatly improved, providing reliable support for accelerating the process of traditional experimental methods. By integrating computational and experimental approaches, these solutions collectively address AF3’s limitations and improve its applicability to complex biological systems.

As AF3 technology advances, we expect it will play a pivotal role in addressing more unresolved challenges and bringing greater breakthroughs to the plant sciences and agriculture.

## Supplementary Information

Additional file1 (PDF 203 KB)

## Data Availability

Not applicable.

## Abbreviations

AF2: AlphaFold 2
AF3: AlphaFold 3
AFDB: AlphaFold protein structure database
BFE: Binding free energy
BRI1: Brassinosteroid insensitive protein
cryo-EM: Cryo-electron microscopic
CASP: Critical Assessment of Structure Prediction
ChIP-seq: Chromatin immunoprecipitation
CIF: Crystallographic information file
cMDH: Cytosolic malate dehydrogenase
FABPs: Fatty acid-binding proteins
ipTM: Interface predicted template modelling
MD: Molecular dynamics
MSA: Multiple sequence alignment
PAE: Predicted aligned error
pLDDT: Predicted local distance difference test
PDB: Protein data bank
pTM: Predicted template modelling
RMSD: Root-mean-square deviation
sHSPs: Small heat shock proteins
3D: Three-dimensional
Y2H: Yeast two-hybrid
