# Supplementary material for: Analysing protein complexes in plant science: insights and limitation with AlphaFold 3
Source: Bot Stud. 2025 May 22;66:14. doi: 10.1186/s40529-025-00462-2 (PMC12098255; doi:10.1186/s40529-025-00462-2)
Supplement: Supplementary file 1 — Additional file1 (PDF 203 KB) [file 40529_2025_462_MOESM1_ESM.pdf]

Additional Information

Supplementary Note1. AF3 not only makes one prediction once at a time. For each job, AF3 returns five predictions per seed but only the top-ranked prediction is shown on the AlphaFold Server result page. The rest four predictions can be accessed through the download button. In addition to one Crystallographic Information File (CIF) for molecular structure visualisation, AF3 provides two output JSON files for each prediction. One describes the relationship between atoms, tokens, and chains; the other one contains the "ranking_score" for ranking the full predicted complex. It incorporates structure confidences such as ipTM and pTM, along with penalties for clashing atoms and disordered fractions. Additionally, the JSON file also provides the confidence metrics of each chain or chain-pair. Users can rank the full complex or the specific chain and interaction based on this information, as higher scores indicate better predictions. In conclusion, AF3 provides multiple predictions per job, along with scores to rank both the entire complex and each component.

Supplementary Note2. The prediction accuracy was quantified by computing RMSD between aligned atom pairs from two structures. RMSD values below 2 Å are generally considered to indicate high structural similarity between computational prediction and experimental structure (Maiorov and Crippen 1995). The RMSD of predicted full-length SKOR D312N/L271P was 30.321 Å, suggesting extremely low similarity with PDB structure and therefore deviation from experimentally validated results, while the domains only shows RMSD of 1.353 Å.

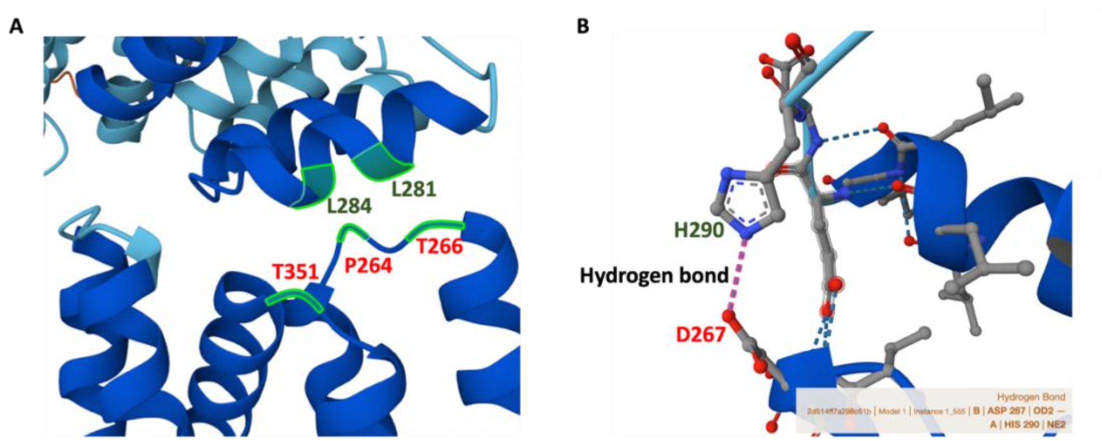

Supplementary Figure 1. Known interacting sites of NPR1-TGA3 in AF3 prediction.

(A) Main residues of interacting site are labelled, residues of NPR1 are in green and residues of TGA3 are in red. (B) Hydrogen bond between residues H290 of NPR1 and D267 of TGA3.

References

Maiorov VN, Crippen GM (1995) Size-independent comparison of protein three-dimensional structures. Proteins Struct Funct Bioinforma 22:273–283. https://doi.org/10.1002/prot.340220308
